# Criminal Behavior and Psychosocial Risk Factors in Brazilian Adolescent Offenders: An Exploratory Latent Class Analysis

**DOI:** 10.3390/ijerph181910509

**Published:** 2021-10-07

**Authors:** Lais Sette Galinari, Marina Rezende Bazon

**Affiliations:** Department of Psychology, Faculty of Philosophy, Sciences and Letters at Ribeirao Preto, University of Sao Paulo, Ribeirao Preto 14040-900, Brazil; mbazon@ffclrp.usp.br

**Keywords:** young offenders, juvenile delinquency, latent class analysis, psychosocial profiles

## Abstract

Considering that adolescent offenders are quite a heterogeneous group in terms of behavioral and psychosocial variables, it is considered that a typological approach can assist in the systematization of these differences, aiming at a better understanding of the phenomenon and at clearer guidance in terms of interventions. Therefore, this study aimed to identify the behavioral and psychosocial profiles of male adolescent offenders, based on empirical data collected in a Brazilian sociocultural context. The profiles were made with a sample of 400 adolescent offenders to perform an exploratory latent classes analysis. The instruments used in data collection were the Youth Behavior Questionnaire (Questionário de Comportamentos Juvenis, QCJ) and the Brazilian Jesness Inventory—Revised (Inventário Jesness-Revisado-Brasileiro, IJ-R-Br). The most appropriate model was that of four classes, with the profiles found indicating differences between the adolescent offenders both in relation to psychological functioning and criminal pattern, as well as the psychosocial risk/protective factors associated with each of the profiles. These findings, in addition to contributing to understanding the phenomenon, may help to reflect on the assessments required to assist in judicial decision-making processes and the customized proposal of psychosocial interventions.

## 1. Introduction

International and Brazilian rules advocate a differentiated justice system for the treatment of adolescent offenders. Such a justice system should take into account the fact that adolescence is a phase of human development in which an individual is subjected to rapid and simultaneous transformations at the physical/biological level—hormonal and neuronal—and at the psychological/personality level, as well as being subjected to new and more challenging social expectations in relation to their behavior [1,2,3]. For these reasons, it is understood that adolescents are a segment vulnerable to the manifestation of deviant behaviors, while also being highly susceptible to behavioral changes due to their great neuropsychological plasticity, which favors psychosocial interventions [4,5]. These regulations propose that juvenile justice must therefore not only hold adolescents accountable but promote their psychosocial development through educational/therapeutic monitoring, which focuses on their social reintegration and on the reduction in the chances of recidivism [1,2,3,6].

Previous research emphasized that educational and/or therapeutic interventions should focus on “criminogenic needs”, that is, personal aspects (impulsiveness, substance use, antisocial values and attitudes) and micro-social aspects (weak family bonds, inappropriate parenting practices, low school performance, offending peers, unstructured routine). These aspects represent dynamic risk factors for the persistence of criminal behavior in adolescents [7,8,9].

However, there is evidence that adolescents who are in the juvenile justice system constitute a heterogeneous group in terms of the problems presented, both regarding the pattern of deviant behavior and in relation to their criminogenic needs [10,11,12]. Thus, the effectiveness of the interventions in this field is conditioned by the adequacy of the contents and the intensity of an intervention towards the characteristics and needs of the target population, i.e., to the customization of the educational/therapeutic monitoring offered to adolescents [13,14].

In different sociocultural contexts, typology studies aiming at the identification of profiles help to better understand the phenomenon of juvenile delinquency [15,16,17], as well as to establish general guidelines for the implementation of programs that are sensitive to intra-group differences [18,19,20]. Typology studies on juvenile delinquency generally report the existence of differentiated profiles, one of which almost always concerns individuals who have a minor criminal behavior, and others concerning individuals who gradually present more severe criminal behaviors. The different patterns of conduct identified refer to differentiated behavior development paths in terms of frequency and severity of the crimes committed, as well as their stability over time, or duration, with each one related to different sets of risk and protective factors [15,16,17,21].

In a literature review carried out by Galinari and Bazon [22] on typology studies in juvenile delinquency, the authors highlight, for example, the recurring identification of a profile related to a pattern of criminal behavior characterized by a high frequency of crimes but of low severity, generally associated with negative social problems—such as poor parenting, low academic performance and criminal socialization—with no marked impairment in personal functioning. They also highlight that, as a rule, a profile related to a more serious pattern of conduct is identified, which involves violent crimes, usually associated with social issues, such as conflicts in the family and at school, as well as certain personal characteristics, such as impulsiveness, anxiety and depression. A third profile, in general, emerges in the reviewed studies and this is related to an even more serious pattern of conduct in terms of frequency, duration and seriousness of the crimes committed. This path is characterized by the early manifestation (before adolescence) of many behavioral problems, associated with family and school issues, and it also appears early in life and displays personal characteristics such as impulsiveness, self-centeredness and a strong antisocial orientation.

It is worth highlighting that latent class analyses have stood out as some of the most appropriate strategies for the creation of a typology, with the identification of profiles remaining a tried strategy [23,24,25,26]. This method of analysis began to be increasingly used in the field of human sciences, especially in terms of health, to find subgroups in a population in terms of relevant variables for intervention strategies [27]. By grouping together the similar, as opposed to the dissimilar, this method allows for the creation of a typology. It certainly does not account for reality in its entire complexity and, sometimes, the established “types” present, between each other, fragile limits based on less reliable criteria than would be ideal [28]. The validity of the typology is all the greater the more it is based on relevant variables, which are ideally based on an empirically grounded model or theory [29].

In this paper, the social and personal control theory of deviant behavior (SPCTDB) was adopted as framework [30,31]. This is a theory that integrates, in a systemic perspective, social and personal variables related to the development of deviant behavior, and it is one of the theoretical models mentioned by Brennan and Brietbach [32] as being compatible with taxonomic methods. The SPCTDB describes the existence of different paths of deviant or antisocial behaviors, and of criminal behavior specifically, among adolescents, as well as their relationships with different social and personal variables. The paths refer to a greater or lesser engagement in the practice of crimes, measured from the pattern of conduct described. In order to measure this pattern, it is necessary, as a general rule, to use self-reported data regarding the age at which the behaviors of interest started, their frequency (total approximate number of behaviors manifested in a determined period of time in the past year, for example) and their diversity (total approximate number of the types of behaviors manifested). In this context, the implication of violence can also be observed [33].

From the perspective of the SPCTDB, criminal behavior in adolescents, with regard to social variables, is regulated based on the interaction between variables related to the following mechanisms: (1) Bond with society and its members; (2) constraints exercised by social institutions and people in the face of deviant/antisocial behavior; and (3) social models related to exposure to antisocial influences and opportunities (as opposed to pro-social ones) [30]. In this study, the privileged variables in this level were the following: low family investment, low parental attachment, criminalized models in the family, family violence, low school bond, low academic performance, behavioral problems at school, expulsion from school, unstructured routine and offending peers. According to the theoretical model, this set of variables related to an adolescent’s life within their family, their school and their relationships with peers of the same age and routine encourages criminal behavior due to the low constraining power exercised by these social institutions with respect to deviant behaviors, due to the fragile bond with them, which results from the weak connection of a given adolescent to the people in these institutions and, at the same time, their exposure to antisocial models and opportunities.

With regard to personal regulation, the theory indicates the importance of psychological development as a mechanism for regulating behavior (the development of allocentrism, as opposed to the typical idiocentrism of the early stages of development), highlighting their capacity for self-regulation/self-control, in view of decreased impulsiveness. Considering the multidimensionality of the self-control construct proposed by Morizot and Le Blanc [34], variables chosen for research that are reported herein were impulsiveness, antisocial values, negative emotionality, and emotional reactivity. It is reiterated that the theory, due to its systemic and dynamic character, presupposes that social and personal mechanisms are related; in other words, that they are inter-influenced [30,31].

Thus, the general objective of this research was to establish a typology through latent class analysis, with an empirical basis, related to patterns of criminal behavior and psychosocial variables in male adolescents in conflict with the law in a Brazilian sociocultural context, as well as to consider the probable existence of different profiles within this population. No research study with this purpose has been identified in the Brazilian sociocultural context. Therefore, the proposal, in addition to seeking to assist in the production of scientific knowledge in a broader way, can serve to validate the profiles described in research studies carried out in other contexts/societies and to denote the weight of sociocultural variables. Thus, it can specifically contribute to the knowledge produced with this type of approach in Brazil, offering elements for reflections around possible profiles of adolescents in conflict with the law and, therefore, the need for differentiated interventions.

## 2. Materials and Methods

### 2.1. Participants

This research was conducted with a convenience sample composed of 400 male adolescent offenders aged 16 years old or older. The option of working only with male adolescents was due to greater accessibility to them, as they are significantly more numerous in the juvenile justice system, and also because the psychosocial variables privileged in the study have been tested for males [35]. Working with data from a mixed sample would therefore generate a confounding effect. In addition, the option to work only with participants aged 16 years old or over was due to the fact that one of the central variables in the study was the participants’ patterns of criminal behavior, which are best apprehended from the second half of adolescence [36]. The mean age of the participants was 16.98 years old, with a standard deviation of 0.78 and the following distribution: 16 years old—27%; 17 years old—49%; 18 years old—24%; 19 years old—1%.

The participants were recruited in programs for the enforcement of court orders. In the city where the study was carried out, and in the period in which data collection was implemented, there were approximately 310 vacancies in detention units, 100 vacancies in provisional detention units and 300 vacancies in community measures, totaling approximately 710 vacancies in the system for the monitoring of adolescent offenders. Therefore, the sample consisting in 400 young individuals refers to nearly 55% of the total adolescents in the system. However, the sample does not proportionally represent the population of the contexts in which the young individuals were recruited, since 84% of the adolescents who participated in the research were in detention and provisional detention units.

Regarding the number of socio-educational measures completed previously, 41.5% of the adolescents reported that they were complying with the socio-educational measure for the first time; 23.5% reported it being their second; 15.5%, noted it was their third; 10%, claimed it was their fourth; and 9.4% said it was their fifth measure (or more). Therefore, the majority stated being repeat offenders in the juvenile justice system (58.5%) and would be in freedom-restrictive measures (84%) aimed, according to the Brazilian law, at adolescents accused of more serious and/or repeat offenses [6]. Therefore, it is to be noted that this study refers, more precisely, to the identification of a typology of adolescents whose cases are considered “more serious” in the socio-educational system.

In relation to the estimate of the mean monthly income per household, obtained by assessing the socioeconomic class according to the Brazil Criterion [37], the following was found, in BRL: BRL 20,888.00—1%; BRL 9254.00—1%; BRL 4852.00—1%; BRL 2705.00—37%; BRL 1625.00—33%; and BRL 768.00—18%, which indicates a lower concentration in the higher classes and a higher concentration in the lower classes. Regarding schooling, we have the following distribution in relation to years of study: 4 years—1%; 5 years—2%; 6 years—7%; 7 years—13%; 8 years—22%; 9 years—23%; 10 years—10%; 11 years—7%. When comparing this distribution with the participants’ age, we found that an important proportion of the sample lagged behind in school.

### 2.2. Instruments

We chose the instruments according to their relevance regarding the privileged variables that were inherent to the mechanisms for regulating adolescents’ criminal behavior; these instruments were therefore relevant in understanding the different paths of development of criminal behavior in adolescents, according to the SPCTDB framework. Consequently, two instruments were employed: The Youth Behavior Questionnaire (QCJ) and the Brazilian Jesness Inventory—Revised (IJ-R-Br).

The QCJ is a version of the instrument originally developed by the Juvenile Delinquency Observatory of the Criminology School at the University of Porto, suitable for use in the Brazilian context. This instrument aims to collect data on criminal behavior in adolescence; it is able to describe behavioral patterns and the personal/behavioral and social/contextual aspects that are related to relevant factors associated with juvenile delinquency. This instrument is similar to that used in the Second International Self-Reported Delinquency Study (ISRD-2) [38]. The questionnaire consists of 56 questions aligned to the variables comprehended by the aspects considered relevant to the social and personal control theory of deviant behavior. The instrument is subdivided into five dimensions according to the following aspects: (1) Adolescent (personal): age, schooling, religion, attitudes and moral values; (2) deviant or antisocial behaviors: divergent behaviors (skipping classes, using alcohol, using marijuana and other drugs, driving a motor vehicle) and criminal behaviors (some without interpersonal violence, such as drug trafficking, handling stolen goods, damage, feud, theft, and others characterized by a violent approach, such as bodily injury, bodily injury with an instrument and theft); (3) family: number of people with whom they live, socioeconomic level, parental bond, investment and supervision (e.g.,: “How often do you play or practice sports with your parents?”, with answers on a scale with four levels: “rarely”, “few times”, “often” or “always”); (4) school: academic performance, school stress, values with respect to studies, school delay (in years) (e.g.,: “The school teaches me things that will help me in the future”, with the answers also being on a scale with four levels: “I totally disagree”, “I disagree”, “I agree”, “I totally agree”); and (5) routine and friends: activities and places frequented during their free time, and friends (pro-and/or antisocial).

The psychometric qualities of the QCJ in the Brazilian sociocultural context were described by Komatsu, Costa, Salgado and Bazon [39], based on a sample of 836 male students attending public and private schools (aged between 11 and 18 years old). The Cronbach’s alpha values ranged from 0.23 to 0.89 and the intra-domain correlations were significant in 81% of the cases, with coefficients ranging from 0.10 to 0.54. The correlations between the scales and the externalizing behaviors were significant in 62% of the cases, ranging from 0.10 to 0.43.

The measure with lower Cronbach’s alpha used was family investment (0.23), while the other Cronbach’s alpha measures were between 0.57 and 0.83. Even though using a measure with a low Cronbach’s alpha may be problematic, as it could reflect a lower reliability, family investment measure was used due to its theoretical importance. Regarding correction of the instrument, the constructs offer raw scores first. Subsequently, these raw scores are transformed into T scores. This instrument presents standards, based on the T score, calculated based on data obtained from the 836 adolescents in the population. For this study, the answers to the instrument were corrected considering the normative data in three different age groups (16 years old, 17 years old and 18 years old or more). In the correction, data from the reference sample, normative and related to students from public schools (*n* = 268), were also considered, seeking a better socioeconomic equalization between the studied adolescents and that of the reference sample. In Brazil, the type of educational institution (public or private) is a good indicator of socioeconomic level, with public schools mostly serving young individuals from less privileged classes.

The IJ-R-Br [40,41] is an adapted and validated version of the Jesness Inventory—Revised [40] for Brazil. It consists of 160 affirmative sentences, to which the adolescent must answer true or false. Different combinations among these items provide scores on 12 different scales, the scores being standardized by comparison of the T score. The mean T score is 50 and the standard deviation is 10 [41]. The instrument assesses opinions/thoughts/beliefs, attitudes, perceptions/distortions, feelings/emotions, psychological defenses, and also some personality traits associated more with criminal behavior, such as impulsiveness, search for sensations and hostility [41]. The instrument’s scales and its respective Cronbach’s alpha indexes obtained from a validation with a sample of Brazilian adolescents are as follows: social maladjustment (SM; α = 0.86), orientation of values (OV; α = 0.82), impulsiveness (Im; α = 0.72), autism (Au; α = 0.75), alienation (Al; α = 0.70), manifests aggressiveness (MA; α = 0.81), withdrawal/depression (Wi; α = 0.70), social anxiety (SA; α = 0.54), repression (Rep; α = 0.42), denial (Dn; α = 0.65), behavioral disorder (BD; α = 0.73) and challenging oppositional disorder (COD; α = 0.70). The IJ-R also generates a score on the associability Index (AI), a measure associated with the risk of recidivism that is calculated from a combination of the scores of SM, OV, Au, Al, MA, Wi, SA and Rep [40].

### 2.3. Procedures

To carry out the research, steps were taken regarding the national and international ethical standards in this field, and the project was submitted to and approved by a Research Ethics Committee (Faculty of Philosophy, Science and Letters of Ribeirão Preto, process number 77903617.5.0000.5407). Before conducting the research, a free and informed consent form was presented to the participants aged 18 years old or over and a free and informed consent form concerning participants under 18 years old was handed over to the parents/guardians, in parallel with the delivery of assent form for adolescents under 18 years of age. First of all, judicial authorization was obtained to collect data from the adolescents in freedom-restrictive socio-educational measures.

Data collection was conducted by applying the instruments. This took place individually, in a private room, within the scope of the programs for carrying out the measures for which the adolescents were recruited, while negotiating the times and conditions for the researcher’s permanence in the place. Considering the fact that many adolescent offenders are not proficient in reading, the instruments were applied in the form of a structured interview (oral format) in order to standardize collection procedures, and thus data reliability. The duration of the individual interviews varied from one hour to an hour and a half with each adolescent.

The data collected were stored in a digital spreadsheet and corrected according to the instruments’ technical standards. A latent class analysis was performed with binary variables from the QCJ and the IJ-R-Br. The gross scores of the QCJ and the IJ-R-Br were standardized using the T score, taking into account the ages (16 years old, 17 years old, 18 years old or more), and classified in a binary fashion; in other words, to establish whether they presented scores above the standard. Scores above 60 were considered as above the T score standard. This procedure aimed at facilitating the analysis of both instruments together and at allowing us to quickly identify the scores of each adolescent in each construct, and to establish whether they were above the normative range while also considering the adolescents in the population of the same age. Aiming at a parsimonious model, in order to define the variables to be inserted in the model, correlation analyses were performed between all the variables available from the QCJ and IJ-R-Br instruments and between those that had a high correlation with each other; one of them was excluded from the analysis. In order to define which would be excluded, care was taken to analyze the theoretical meaning in order to preserve those most aligned to the SPCTD model.

Thus, the LCA was performed with the following variables: (a) Pattern of criminal behavior—diversity of crimes; frequency of crimes; diversity of violent crimes; (b) social/contextual variables—low family investment; low parental attachment; low school bond; behavioral problem at school; unstructured routine; offending peers; (c) psychological variables—social maladjustment, low denial, repression, social anxiety, alienation and manifests aggressiveness. Thus, the latent class analysis was performed with R Software using the “poLCA—Polytomous Variable Latent Class Analysis” package. To choose the ideal number of classes, models from one to seven classes were generated and the AIC and BIC criteria were used to compare the fit and choose the model that best represented the sample data.

The choice of model, in terms of the ideal number of classes representing the sample data, was made by comparing the different models based on fit statistics, which were generated at the time of the analysis. In addition, the consistency of each model was also assessed by analyzing the theoretical meaning of the classes inherent to the different models generated [40]. We decided to use the BIC (Bayesian Information Criterion) fit criterion, since there was evidence showing it to be more suitable for more complex models, with greater robustness and success rates [42,43]. After identifying the classes, they were compared using a chi-square test regarding their distribution with respect to the categorical variables: socioeconomic class, daily use of marijuana (yes/no), low academic performance (yes/no), expulsion from school (yes/no), criminalized models in the family (yes/no) and episodes of family violence (yes/no).

## 3. Results

**Figure 1:**
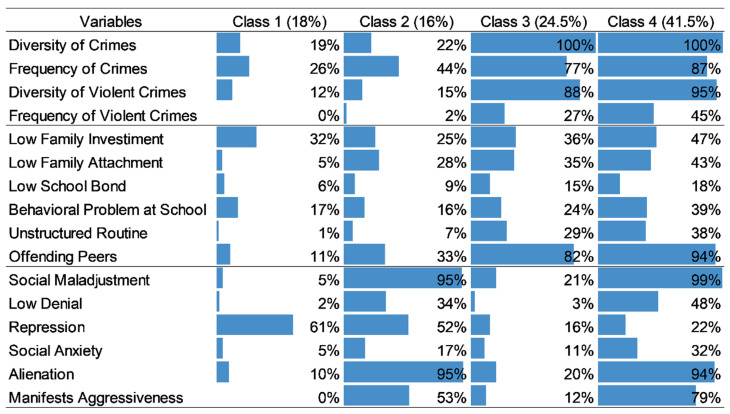
Percentage of adolescents from each class who obtained an “above the standard” score in the model variables.

The model chosen was that comprising four (*n* = 4) classes, since it was the one with the lowest BIC and the highest consistency with respect to the data (BIC: 6814.43; AIC: 6547.01; chi-square goodness of fit: 57,487.11). Figure 1 shows the result of the latent class analysis. The classes identified were ordered based on the pattern of criminal behavior: least severe (Class 1) or most severe (Class 4) criminal behavior. The results around the variables were ordered in relation to social dimensions (involving the variables that refer to social regulation) and personal dimensions (involving the variables that refer to the personal regulation of behaviors, related to psychological functioning).

In synthesis, it can be considered that Class 1 (C1) contains adolescents who most resemble those of the general population. Most of them presented scores close to the standards regarding the variables that describe patterns of criminal behavior, denoting, for all, an absence of implication in violent crimes. With regard to the social/contextual and psychological variables, the scores of the majority were also close to the standard, except in repression, where the majority (61%) presented a score above the standard. High scores on this IJ-R-Br scale indicate difficulty in naming and identifying negative feelings and emotions, such as anger and disgust [41].

Class 2 (C2) comprised adolescents who, for the most part, present predominantly normative scores with regard to criminal behavior (although a considerable part—44%—showed frequency of crimes above the standard). Regarding social/contextual variables, the majority were also within the standard, highlighting the fact that only 33% had offending peers above the standard. In terms of psychological variables, however, the majority had scores above the standard in four of the variables: social maladjustment, indicating antisocial attitudes and impulsiveness above the standard; repression, indicating difficulty in naming and identifying negative feelings and emotions, also above the standard; alienation, indicating difficulty in identifying the other/having empathy, above the standard); and manifests aggressiveness, indicating feelings of anger and emotional discomfort above the standard [41].

Class 3 (C3) included adolescents who, as a majority, presented a pattern of criminal behavior characterized by frequency and diversity of crimes above the standard, including diversity in violent crimes, even though their frequency, for most of them, was within the standard. However, the majority did not present frequency of violent crimes above the standard. In relation to the social/contextual variables, most presented an association with offending peers above the standard. It is noteworthy that nearly one third (or more) had scores above the standard, as well as in relation to low family investment, low parental attachment and unstructured routine. Regarding the psychological variables assessed, C3 adolescents, for the most part, present normative functioning.

Regarding Class 4 (C4), it is easier to indicate the variables in which the majority did not obtain scores above the standard. The scores in low school attachment, repression and, at the limit, social anxiety, did not stand out in this class. In the other variables, between at least 30% and 100% of the sample obtained scores above the standard. With respect to behavior, all the adolescents (100%) presented scores above the standard in diversity of crimes. Most presented such scores in frequency of crimes in the past year (87%) and in diversity of violent crimes (95%). In addition, a considerable proportion (45%) presented frequency of violent crimes above the standard. As for the social/contextual variables, more than one third of the adolescents presented scores above the standard in low family investment (47%), low parental attachment (43%), behavioral problems at school (39%) and unstructured routine (38%), with the vast majority (94%) presenting a score above the standard in offending peers. Regarding the variables of psychological functioning, almost all of them presented non-normative scores in antisocial attitudes and impulsiveness (99% in social maladjustment), in strangeness and difficulty in identifying other (94% in alienation) and in feelings of anger and emotional discomfort (79% in manifests aggressiveness). More than one third (48%) presented a non-normative score in low denial (48%). Denial, by itself, refers to the capacity of individuals to remain optimistic, even in the face of difficulties. Low denial, therefore, indicates the opposite, i.e., it denotes skepticism and pessimism [41].

Table 1 synthesizes the characterization of the classes in relation to family income ranges and in relation to the binary variables related to the use of marijuana (daily use), low academic performance, expulsion from school, criminalized models in the family and episodes of violence, as well as the result of the chi-square test.

It is noteworthy that there was no evidence of differences between the groups regarding the distribution in relation to the economic income of the families represented in each class (X^2^ = 0.178; *p* = 0.271). A significantly lower proportion of adolescents in C1 (28%) used marijuana daily in comparison to the other classes. Therefore, in C4, the proportion is significantly higher (55%) (X^2^ = 16.39; *p* = 0.001). C3 (26%) and C4 (27%) were the classes with the highest proportion of adolescents with low academic performance (X^2^ = 7.95; *p* = 0.047) and also with the highest proportion of adolescents who reported having been expelled from school (29% for C3 and 44% for C4) (X^2^ = 25.96; *p* = 0.000). The proportion of adolescents who reported having criminalized models in the family in C1, C2 and C3 was similar (between 63% and 69%), while, in C4, it was significantly higher (81%) (X^2^ = 7.814; *p* = 0.05). In addition, in this class, the proportion of adolescents who reported episodes of family violence was also significantly higher (44%) (X^2^ = 25.46; *p* = 0.000).

## 4. Discussion

The analysis carried out allowed for the identification of subgroups related to patterns of criminal behavior and psychosocial characteristics, from which it is possible to infer well-defined profiles, or classes (C1, C2, C3 and C4), composing a typology of adolescent offenders. By adopting a qualitative approach to the interpretation of the data characterizing each profile, we point out that the profile emerging from C1 refers to adolescents who, although judicialized due to some offense, would essentially not be different from adolescents in the general population, either in terms of manifest deviant behaviors or of exposure to social and personal variables that could be considered as criminogenic needs. The only characteristic in this profile that, in comparison with the normative data, would be out of place, is the score in “repression”, one of the variables assessed in terms of psychological functioning. In this profile, therefore, there is an above-the-mean recurrence to a defense mechanism (repression), in the sense of dealing with unpleasant emotions and thoughts that are linked to aspects perceived as negative in oneself and/or in relationships; individuals suffering from repression tend to suppress such emotions and thoughts. Considering a risk and needs approach [7], these youths have low intervention needs. In these cases, an extrajudicial intervention or a judicial intervention with low intensity, such as probation, would be appropriate.

The profile that emerges from C2 refers to adolescents who, despite having more important offense engagement compared to those in C1, show a pattern of criminal behavior characterized by low frequency and non-involvement in violent crimes. Most of them are not exposed to specific risk factors at a social level to a significantly greater extent than the young individuals in the sample of the reference population, except for offending peers. However, in this profile, the most discordant characteristics, when thinking about the standard, are the scores in social maladjustment and alienation, both variables related to problems at the personal/psychological regulation level. These variables denote young individuals with antisocial beliefs, values and attitudes and with mistrust towards others above the mean. In terms of risk and needs [7], youths belonging to C2 need interventions focused on antisocial beliefs, values and attitudes, as well as on attendance at prosocial socialization environments due to the presence of offending peers. Considering the low criminal behavior pattern in C2, less restrictive measures should be approved.

The profile that emerges from C3 refers to adolescents who present greater offense engagement, with a frequency and diversity of crimes well above the mean, as well as practicing violent crimes. In parallel with this pattern of criminal behavior, there are scores above the mean on a wide variety of social factors, especially family violence, notably within the family, and school and peer relationships (they tend to have more offending peers). In this profile, however, difficulties at the personal/psychological level do not stand out, since the scores of most young individuals, in the different variables considered, are in the normative range. Considering a risk and needs approach [7], youths belonging to C3 have intervention needs focusing on improving family and school relationships. Once C3 adolescents present greater offense engagement, perhaps in some cases a more intense measure would be necessary initially.

On the other hand, the profile that emerges from C4 can be considered as the most serious cases, both in terms of offense engagement and exposure to psychosocial risk variables. Similar to what was observed in C3, in C4, adolescents present scores above the mean in frequency and diversity of violent crimes and in a wide range of the social and personal variables assessed. Their scores are above the normative range in low family investment, low parental attachment, behavioral problems at school, unstructured routine and offending peers. In addition, they have significantly more episodes of family violence and more criminalized models in the family. Their scores are also above the normative range in social maladjustment, alienation, manifests aggressiveness and low denial, denoting a profile characterized by a strong antisocial orientation, low impulse control, skepticism, mistrust towards others, negative/anger emotions and resentment. In a risk and needs perspective [7], C4 youths have complex intervention needs. Their intervention needs should focus on psychological characteristics such as antisocial orientation and self-control. They also require intervention needs focused on social aspects, specifically on developing healthier family, peers and school relationships and attending at prosocial environments. Once C4 adolescents present serious offense engagement, it is possible that a more intense and restrictive measure would be necessary initially.

Considering the typologies described in the literature, established in other sociocultural contexts, we may argue that, in many studies, a profile similar to the one that emerges from C1 has been identified. In other words, that of young individuals who have low offense engagement and few or almost no criminogenic needs [15,20]. Typology studies with a longitudinal design have also identified a similar profile, and the data denoted that the adolescents whose data helped to constitute it would have a punctual contact with the juvenile justice system and a low risk of recidivism (or persistence of criminal behavior) [17,36,44].

It is important to highlight that some of these adolescents whose data comprised C1 were in a closed disciplinary regime, which, considering the characteristics highlighted above, would be a disproportionate punishment considering “the intensity of the problem presented”, and therefore harmful to the adolescents in question. According to some authors, the institutionalization process is almost always harmful to adolescents who have this type of classification; in other words, those who present a psychosocial development that is close to normative, due to processes such as criminal socialization and stigmatization which, in general, accompany the closed disciplinary regime [45].

Focusing on C2, some typological studies also describe a profile that presents a pattern of conduct that cannot be considered normative, but which is also not very serious/severe. In these studies, however, this profile is characterized by exposure to more risk factors, such as those related to difficulties in school, which is indicative of social adaptation problems, as well as important uses of psychoactive substances [15,16,18]. In these studies, family neglect also stands out, involving lack of supervision, with a reflection on the development of a young person’s social skills, leading them to be deficient in this area [16,18]. In the profile that emerged in this study, prominent variables are related more to antisocial orientation and distrust of others, as aspects of psychological functioning, which perhaps denote socialization in contexts of exclusion and marginalization, are pervaded by antisocial values and beliefs. In this sense, it is possible that the profile derived from C2 reveals aspects of the Brazilian socioeconomic and cultural reality. This profile is similar to others identified in studies that work with classifications in terms of risk exposure levels, in which one of the profiles is notable for its “moderate risk” for offense recurrence; this is mainly due to some difficulties in relation to personal dimensions (antisocial values/attitudes) [23,46,47].

The C3 profile is y similar to others described in the literature, and it also presents a pattern of serious criminal behavior that is generally associated with experiences of conflicts in relationships with significant figures in social institutions, such as family and school, without, however, evidencing a clear/strong antisocial orientation [15,16,18]. Thus, such a profile would be less dependent on particular sociocultural characteristics and, perhaps, more linked to relational problems that are related to intergenerational conflicts [47,48]. It is worth emphasizing that this profile, identified in different studies with different samples and methodologies, denotes the existence of a serious juvenile delinquency that is not based on a relevant antisocial orientation or derived from antisocial socialization, which supports the proposition that the phenomenon of persistent juvenile delinquency can have different etiologies. For example, in a study by Decuyper et al. [49], one of the identified profiles was characterized not by presenting a marked antisocial orientation but rather by traces of impulsiveness that result in disruptive behavior and, consequently, in conflicts. Negative/coercive parenting practices and family violence, abuse and neglect, as well as school conflicts are common [47,48], as well as mental health issues such as anxiety, depression and psychological malaise [15,16,18]. These aspects were not investigated in the study reported here, which can be considered as a limitation.

As for C4, a parallel can be made with the profiles identified that represent adolescents with a more serious pattern of criminal behavior that is linked to exposure to many social and personal risk factors. In this study, based on the variables differentially present in the profile perceived in C4, there is a strong antisocial orientation and a weak social bond. Problems in the family and at school, in addition to personal characteristics such as impulsiveness, aggressiveness, egocentrism and low self-control, are at the basis of the development of criminal behavior, while also taking into account associations with offending peers regarding the context of criminal socialization. A characteristic almost always present in the characterization of profiles similar to the one described in C4 is the presence, at the psychological level, of so-called psychopathic characteristics, such as superficial charm and a tendency to manipulate others for their own benefit [15,16,18]. However, these aspects were not investigated in this study.

We highlight that over 40% of the adolescents were classified in the most serious offending class (C4), and most previous typologies do not usually have such high proportion of serious offenders [15,16,18,21]. We hypothesize that this difference can be explained by the fact that most participants (84%) were in detention and provisional detention units intend for “high risk youths”. The presence of C1 and C2 adolescents in those contexts reveals a serious assessment failure by the Brazilian juvenile justice system.

Studies that work with classifications in terms of risk exposure levels indicate that this profile has a “high risk” for offense recidivism [21,48]. Previous longitudinal research also indicates that such a profile is associated with individuals whose criminal behavior presents a greater chance of continuing beyond the adolescence years [17,36,50].

Despite the fact that the profile derived from Class C4 refers to more “serious” delinquency, it is emphasized that most of the variables integrated into the creation of the typology have a dynamic character. Thus, the possibility of promoting changes in paths, through appropriate psychosocial interventions, must always be underlined. As indicated by Walker et al. [48], it is true that profiles that are characterized by a larger number of problems require treatment with a multidimensional approach. When carrying out a meta-analysis on the effectiveness of intervention programs for adolescent offenders, Lipsey [51] identified that adolescents classified as having a “high risk” for recidivism, linked to several social and personal factors, were those who benefited the most from multimodal programs, with a greater reduction in recidivism rates.

The results suggest that factors that recurrently appear to be associated with more serious criminal behavior patterns are important targets in terms of primary and secondary deviance prevention, such as family and school bonds, offending peers’ association, antisocial values and low self-control. We highlight that the common factor between C3 and C4 (classes with more serious behavior pattern) is offending peers. Thus, this factor could be understood as an important criminogenic variable. On the other hand, both C2 (with a less severe behavior pattern) and C4 (with the more serious behavior pattern) present score above the standard in social maladjustment and alienation. We could hypothesize that those variables are not always a criminogenic factor, in spite of their importance for differentiating types and indicating intervention needs.

Finally, we highlight that the only characterization variable in which there was no evidence of significant differences between the classes identified in this study was socioeconomic class. Although belonging to a disadvantaged socioeconomic class can be a vulnerability in the sense of enhancing the effects of specific factors, Le Blanc [31] indicates that it cannot be considered as a determinant of criminal behavior. For the author, proximal variables, such as social bonds, and certain personal aspects, such as impulsiveness/low self-control, are more important. In a study by Galinari, Vicari and Bazon [52], socioeconomic class represented, in an adjusted model of multiple logistic regression, an important factor for the judicialization of adolescents (which does not correspond to the phenomenon that was privileged in our study: offense engagement).

## 5. Conclusions

It is important to point out some limitations of the research in question, as they affect the generalization of our results. The first limitation to be considered refers to the sample composition. The socio-educational system recommends six different types of penalties, and the adolescents in the sample were predominantly in detention and provisional detention units. Considering that these are the most freedom-restrictive measures, it is possible to hypothesize that a similar study with a more diverse sample, with greater representation of the young individuals in the socio-educational system as a whole, would identify greater concentration of adolescents in classes with a less severe pattern of criminal behavior, contrary to what was identified in this study. Surveys carried out in the Brazilian sociocultural reality indicate that the percentage of young inmates varies widely in the different states of the federation, with the state of São Paulo being third in the country with the highest rate of young inmates [6].

In addition to that, considering that a typology should serve, above all, to help identify and understand different intervention needs, we emphasize that this study was carried out by taking into account a limited number of variables, excluding some that could provide information related to important risk factors. One of the variables that was not studied was mental health, for example. In this context, that this study did not include research on some protective factors. The emphasis on risk factors generates risk profiles, which could be different if, in the clustering, aspects that can moderate the risk were taken into account. Craig, Piquero and Farrington [12] verified that high verbal IQ, school attachment, low hyperactivity, high parental engagement and adequate supervision were shown to be predictors of success in adulthood for individuals who, as children, were classified as belonging to a group at a high risk for delinquency.

The research, however, provided contributions to scientific knowledge. The study confirmed the heterogeneity inherent to the group of adolescent offenders, even among those subjected to the same judicial measures, whether with regard to the self-revealed pattern of deviant behavior or the associated psychosocial variables. In addition, it corroborated that a pattern of persistent criminal behavior can be associated with different sets of factors, which contributes to underlining the importance of some customization in the provision of services to adolescents in the adjustment of the treatment programs to the profiles in question [15,20]. Conceiving and treating offenders in a similar manner, as if they were a homogeneous group, or making considerations about the problems and needs of each young person in an intuitive way, without the support of any form of systematization, is absolutely unacceptable, as is clear from the evidence provided by several typology studies.

In future research studies, considering the different profiles that are linked to different intervention needs, it is important to propose, implement and evaluate intervention protocols that consider this heterogeneity in adolescent offenders, in order to verify effectiveness levels profile by profile. Furthermore, in relation to future research studies, the method should be replicated with larger and more representative samples, adding variables related to mental health problems and protective factors in order to identify a more complex typology, and one that is closer to the reality of the phenomenon of juvenile delinquency. In addition, it is also important to replicate the study with an independent sample of adolescent offenders who are in the closed prison regime. Doing so would allow us to assess whether the results we found are confirmed.

## Figures and Tables

**Table 1:** Characterization of the classes in relation to the categorical variables and to the chi-square test.

	Class 1	Class 2	Class 3	Class 4	X^2^	*p*
Estimated monthly family income (BRL)						
20,888.00	0%	0%	1%	1%	0.178	0.271
9245.00	0%	0%	0%	1%		
4852.00	10%	9%	18%	8%		
2705.00	35%	41%	43%	34%		
1625.00	32%	34%	24%	38%		
768.00	24%	16%	13%	18%		
Daily use of marijuana						
	28%	48%	42%	55%	16.39	0.001
Low academic performance						
	11%	19%	26%	27%	7.957	0.047
Expulsion from school						
	17%	17%	29%	44%	25.96	0.000
Criminalized models in the family						
	69%	63%	65%	81%	7.814	0.05
Family violence						
	7%	23%	33%	44%	25.46	0.000

## Data Availability

The data presented in this study are available on request from the corresponding author. The data are not publicly available due to ethical aspects.
